# The complete mitochondrial genome of the Ctenophore *Beroe cucumis*, a mitochondrial genome showing rapid evolutionary rates

**DOI:** 10.1080/23802359.2019.1580165

**Published:** 2019-10-24

**Authors:** Minxiao Wang, Fangping Cheng

**Affiliations:** a CAS Key Laboratory of Marine Ecology and Environmental Sciences and Centre of Deep-Sea Research, Institute of Oceanology, Chinese Academy of Sciences, Qingdao, China;; b College of Marine Ecology and Environment, Shanghai Ocean University, Shanghai, China;; c Centre for Research on the Ecological Security of Ports and Shipping, Shanghai, China

**Keywords:** Ctenophora, mitochondrial genomes, DNA barcoding

## Abstract

We described the complete mitochondrial genome of the Ctenophore *Beroe cucumis*, which is a circular molecule of 10,487 bp in length. The new mitochondrial genome comprised only 12 genes, making it one of the smallest animals’ mtDNA. Both nucleotide substitution and gene order rearrangements exhibited extreme high evolutionary rate in mitogenomes of Ctenophore. The phylogenetic analysis based on mitogenomics failed to reveal the basal position of Ctenophore within metazoan, owing to the extreme evolutionary rate. Based on the available Ctenophora mitogenomes, we found the optimized primers designed by Geller et al. for DNA barcoding suited for the taxon.

## Introduction

Ctenophora (comb jellies) are a small phylum composed of about 200 species (Mills 2017; http://www.marinespecies.org/). Although they possess complex nervous and mesoderm-derived muscular systems, the Ctenophora were believed to be the basal animal lineage (Mcfadden et al. 2011; Moroz et al. 2014). Mitochondrial genomes/genes have been proved to be good tools for studies on molecular evolution and species identification (Geller et al. 2013). However, the under-sampled status of Ctenophora’s mitochondrial genomes made the applications of the marker in trouble. Lack of the mitochondrial genomes complicated the primer design of DNA barcode in the Ctenophora. Here, we sequenced a mitogenome belonging to *Beroe cucumis*.

**Figure 1.**
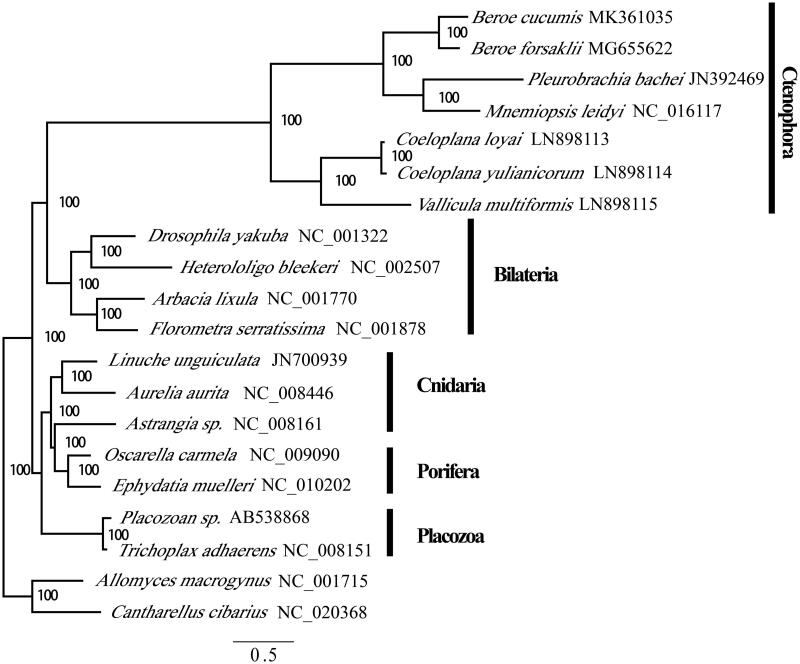
Phylogenetic position of Ctenophora within Metazoan using concatenated proteins in mitochondrial genomes. Numbers at each code are Bayesian posterior probabilities (percentage). GenBank accession numbers of each species were listed in the tree.

The sample of *B. cucumis* was collected in the Jiaozhou Bay (36°06′N, 124°14′E) in August 2016 and preserved under −80 °C in the Center of Deep-Sea Research, Institute of Oceanology, CAS, China (sample code: JZ-20160801BC). The genomic DNA was extracted using Omega plant DNA mini kit (catalog no. D3485-01; Omega Bio-tek, Norcross, GA, USA). The complete mtDNA was obtained by assembling (clc genomic workbench v11) the 10G 150 bp pair-end reads produced by Illumina X10 (Figure 1). The annotated mtDNA was deposited in the GenBank with the accession number MK361035.

The complete mitochondrial genome of *B. cucumis* is 10,487 bp in length and highly AT-biased. The A + T content (81.51%) is similar to other Ctenophora species (Arafat et al. 2018), but higher than typical invertebrate species. The AT-skew and GC-skew of this mtDNA were −0.398 and −0.068 respectively. Like other Ctenophora species, the mitochondrial genome coding genes located on the same strand and lacked all the tRNAs, ATP6/8, and ND6 genes. The PCGs start with ATA (ND2, ND3, and ND5) and ATT (others), and terminated with TAA except for ND2 with TAG. This mitochondrial gene order is identical to that of *B. forskalii*, but different from other Ctenophora. Gene order of Ctenophora was highly rearranged except for those from the congeneric species as reported before (Arafat et al. 2018). The high evolutionary rate (Pett et al. 2011) was confirmed by the relative low similarity between the sibling *Beroe* species based on the blastn comparison results (76% identity for the whole mtDNA and maximum 88% identity for the COX1 gene). Generally, 97% similarity was adopted as the gold standard to distinguish the congeneric species.

We investigated the phylogenetic relationships of ctenophore using the amino acids of mitochondrial genome coding genes. Best partitioning scheme was evaluated in partition finder 2.1. As reported in previous studies, our analysis was unable to resolve the basal position of the ctenophores within metazoans owing to the extreme evolutionary rate (Osigus et al. 2013). In our BI (Mr. Bayes) inferred trees, the Ctenophora were positioned as the sister clade of Bilateria. However, the results confirmed the monophyly of *Beroe* and the high mitochondrial evolutionary rate within Ctenophora.

Finally, we found that the primer-targeted regions of Ctenophora gave a similar level of mismatches compared to other taxa, suggesting the optimized primers for DNA barcoding (Geller et al. 2013) suited for the Ctenophora.
